# Transitional and Long-Term Rehabilitation Care System After Stroke in Korea

**DOI:** 10.3389/fneur.2022.786648

**Published:** 2022-03-31

**Authors:** Ja-Ho Leigh, Won-Seok Kim, Dong-Gyun Sohn, Won Kee Chang, Nam-Jong Paik

**Affiliations:** ^1^Department of Rehabilitation Medicine, Seoul National University College of Medicine, Seoul National University Hospital, Seoul, South Korea; ^2^National Traffic Injury Rehabilitation Research Institute, National Traffic Injury Rehabilitation Hospital, Yangpyeong-gun, South Korea; ^3^Department of Rehabilitation Medicine, Seoul National University College of Medicine, Seoul National University Bundang Hospital, Seongnam, South Korea; ^4^Graduate School of Public Health, Seoul National University, Seoul, South Korea; ^5^Medical Rehabilitation Center, Korea Workers' Compensation Welfare Service Incheon Hospital, Incheon, South Korea

**Keywords:** stroke, rehabilitation, transitional care, long-term care, community health service, Korea

## Abstract

Stroke is one of the leading causes of mortality and disability in Korea. Patients who experience stroke require adequate management throughout the acute to subacute and chronic stages. Many patients with long-term functional issues require rehabilitative management even in the chronic stage. A comprehensive rehabilitation and care model for patients who experience stroke is necessary to effectively manage their needs during rehabilitation and allocate medical resources throughout the stages, thus ensuring reduced unmet needs and improved post-stroke quality of life. In Korea, the government and medical specialists are working on re-organizing the rehabilitation care model, including standardized triage and discharge planning after acute stroke treatment, and establishing systematic transitional and long-term rehabilitation care plans. This review briefly introduces the general rehabilitation triage after acute stroke and describes the current transitional and continuous care systems available for these patients in Korea. We also present the issues faced in transitional and long-term care plans of the current system and the efforts invested in resolving them and promoting long-term care in stroke cases.

## Introduction

Stroke is one of the leading causes of mortality and disability in Korea (1). According to a recent executive summary of stroke statistics in Korea, the incidence of stroke is 232/100,000 persons/year. Mortality due to stroke is gradually decreasing due to early and adequate acute care; nonetheless, stroke remains a public health concern because one in forty adults lives with stroke (1). Approximately 30% of stroke survivors need assistance in their basic daily activities [modified Rankin Scale (mrs) 3 to 5] 3 months after stroke onset; this disability leads to higher costs and worse quality of life (2–4). On including patients who need assistance with their usual duties and activities (mRS 2) or have any stroke-related symptoms (mRS 1), the proportion increases up to 74% (2). Therefore, an adequate individualized rehabilitation plan is mandatory for patients with acute stroke to reduce long-term disabilities and socioeconomic burdens (5, 6). However, the time window for the effect of rehabilitation on functional recovery is limited as survivors reach their functional plateau 6–12 months after stroke onset (7, 8). Thereafter, they must adapt their lives according to their disabilities.

Patients with stroke have long-term medical and functional problems and need adequate care to address these problems. For instance, patients with stroke in Korea may experience worsening problems in multiple domains associated with health-related quality of life (e.g., mobility, spasticity, pain, mood, communication, cognition, and life after stroke) during follow-up (9). In a recent systematic review of 19 studies on long-term unmet needs after stroke, the median number of self-reported unmet needs ranged from two to five in body functioning, activities/participatory, and environmental domains according to the International Classification of Functioning, Disability, and Health (10), and on an average, 73.8% of the patients reported at least one unmet need (11). Among service needs, unmet needs for information, transport, home help/personal care, and therapy were common (11). At 1 year after stroke, approximately 20% of patients reported unmet needs for rehabilitation services (12). Therefore, after patients with stroke are discharged from the hospital, a transitional and long-term rehabilitation care plan is required to reduce the worsening of various functional domains and fulfill unmet needs to improve post-stroke health-related quality of life.

Countries in the Asia-Pacific region have different strategic plans and public health policies for secondary prevention and rehabilitation after stroke (13). In Korea, the regional cardiocerebrovascular center program consisting of four subcenters in each regional center (cardiovascular, cerebrovascular, rehabilitation, and preventive center) was initiated by the Ministry of Health and Welfare (MOHW) of South Korea to promote the nationwide quality of acute care for patients with cardiovascular and cerebrovascular diseases since 2008 (14). A total of 14 regional cardiocerebrovascular centers have been installed. The rehabilitation center provides early and subacute rehabilitation after stroke and further triages patients to transitional or long-term care programs after inpatient rehabilitation. The Act on the Prevention and Management of Cardio-Cerebrovascular Diseases has been enforced since 2017 to alleviate the personal suffering and social burden resulting from cardiocerebrovascular diseases and promote patients' quality of life (15). A comprehensive management plan for cardiocerebrovascular disease from 2018 to 2022 has also been developed by the MOHW. It includes the agenda to promote appropriate and sufficient rehabilitation and continuous care after stroke (16).

This review briefly introduces the general rehabilitation triage after acute stroke in Korea and then describes the current transitional and continuous care systems available for patients with stroke in Korea. We also discuss the problems with the Korean system and the efforts to address problems and promote long-term care in stroke. By reviewing the current protocol in the country, we can learn from each other how to promote long-term care of stroke survivors and plan clinical research to provide important evidence for stroke care policy.

## Rehabilitation After Acute Stroke and Triages

**Figure 1:**
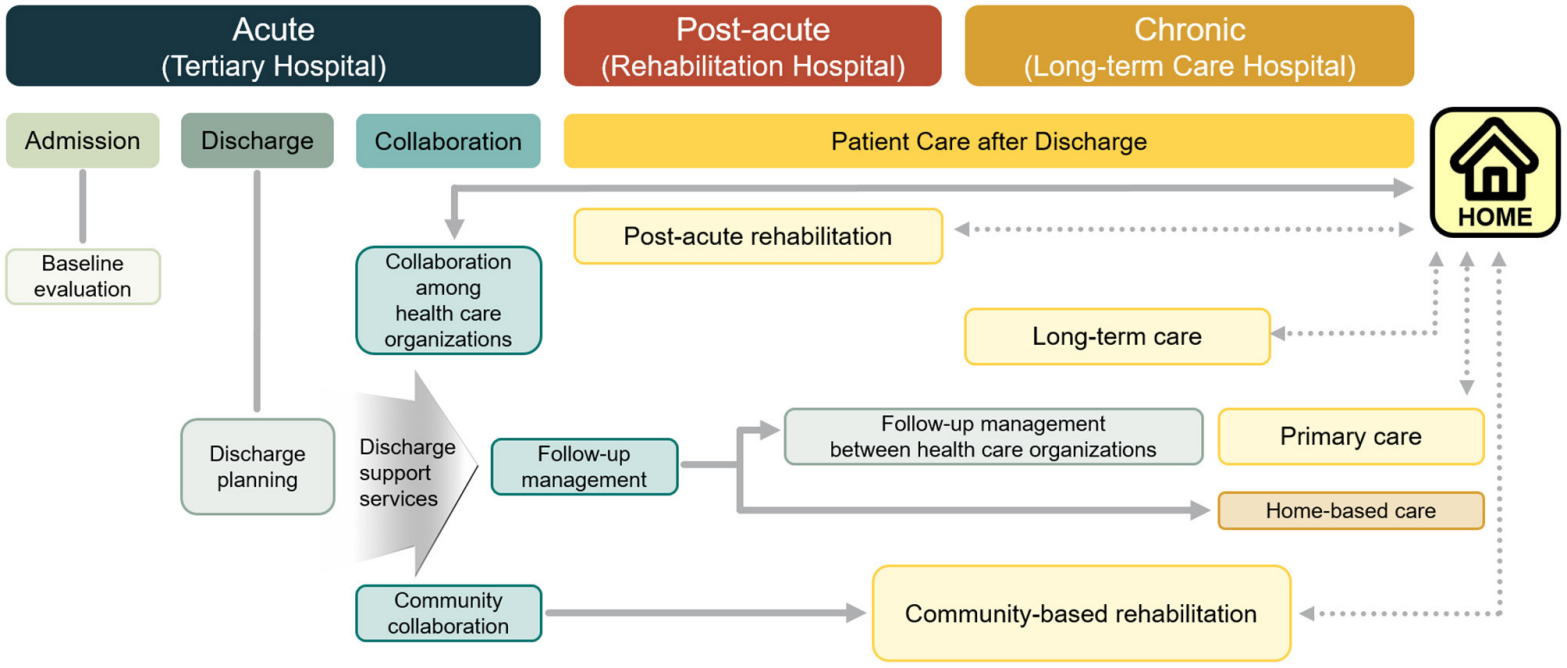
Medical rehabilitation delivery system for patients with stroke.

In a comprehensive stroke center or regional cardiocerebrovascular center, stroke survivors are referred to physiatrists during hospitalization to evaluate the functional ability and rehabilitation needs. According to the initial evaluation for rehabilitation, patients who need comprehensive inpatient rehabilitation are usually transferred to a rehabilitation center (Figure 1). If the patients are not fully recovered and are not ready to be discharged to their homes after short-term intensive inpatient rehabilitation, they are transferred to long-term rehabilitation hospitals. If a patient has a mild impairment and is neurologically and medically stable at the initial evaluation after acute stroke, they can be discharged and can receive outpatient rehabilitation as per need. According to a report from the Korean Brain Rehabilitation Database from 2007 to 2011 (*n* = 5,212 patients from 46 hospitals), the transfer time to the rehabilitation center after stroke onset and length of stay in the rehabilitation center after transfer gradually decreased. The transfer time after stroke onset and median length of hospital stay during 2011 were 30 days and 28 days, respectively (17). The median transfer time after stroke onset was shorter for ischemic strokes (19 days) than for hemorrhagic strokes (35 days) (17). The Korean version of the modified Barthel index gained 18 points during the inpatient rehabilitation period (17). Based on the registry for 11 regional cardiocerebrovascular centers from June 2014 to December 2017, among 17,862 patients with stroke referred for rehabilitation, 3,716 (20.8%) patients were transferred to rehabilitation centers (18). The mean transfer time after stroke onset and length of stay at the rehabilitation center were 10.1 and 22.1 days respectively. Approximately 36% of patients were discharged to home from regional rehabilitation centers (18).

For acute stroke care quality control, the Health Insurance Review and Assessment Service of Korea evaluated nine indicators for care quality grading and fifteen indicators for monitoring in 2018 (19). Among these indicators, there are three rehabilitation-related indicators. The proportion of early assessment for rehabilitation needs within 5 days after admission due to acute stroke is one of the indicators for quality grading; 98% of patients with acute stroke in 242 hospitals received early rehabilitation assessments in 2018. The proportion of patients who received the required rehabilitation during admission among patients assessed as needing rehabilitation in early assessment was 93.7%, and the median number of days from admission to the provision of rehabilitation service was three (19). These results were obtained from the data in tertiary or general hospitals, and it seems that there is a regional discrepancy in these clinical indicators. Therefore, the comprehensive management plan for cardiocerebrovascular disease from 2018 to 2022 in Korea included an agenda to promote early assessments and services for rehabilitation after acute stroke based on the regional and primary cardiocerebrovascular centers (16).

## Transitional and Long-Term Rehabilitation Care System

### Status of Medical Rehabilitation Providers

Institutions that provide inpatient rehabilitation services for patients with stroke in Korea include tertiary and general hospitals where the department of rehabilitation medicine is established. These facilities also provide outpatient rehabilitation services, while community-based rehabilitation is mainly provided by public community healthcare centers and their collaborators (20). Recently, there has been a shift in the focus of rehabilitation for patients with stroke who have completed acute treatment from acute care hospitals to transitional and long-term care hospitals (LTCHs). In the past decade, the proportion of inpatient rehabilitation services provided by LTCHs has continued to increase. In comparison, outpatient rehabilitation services are still provided by acute care hospitals, indicating poor accessibility to rehabilitation for patients in the subacute and chronic stroke phases (21).

In the last 10 years, from 2007 to 2017, the proportion of in-patient rehabilitation services provided by the tertiary hospitals, general hospitals, and other hospitals decreased from 14.8%, 25.3%, and 29.5% to 5.8%, 10.9%, and 28.8% respectively. While the proportion of that provided by the LTCHs surged from 26.7 to 48.0%. For outpatient rehabilitation, the proportion provided by the tertiary, general, and other hospitals increased, and the proportion provided by LTCHs and primary clinics decreased. This result shows that outpatient rehabilitation services for patients with stroke are not yet institutionalized and enforced in Korea (21).

There is no consensus on the hospitalization period for rehabilitation delivery in Korea. The length of stay (LOS) for patients with stroke tends to be longer in Korea than in other countries. According to a study that used the Multicenter Prospective Observation Study data, the mean LOS, including rehabilitation from stroke onset to home discharge, was 115.6 days (median, 19.4 days) with a positively skewed distribution of LOS (22). Another previous study also showed that the mean LOS of patients with stroke in Korea was 191.5 days (23).

Studies have reported LOS after stroke in Spain (64.1 days), in the United States [16 days (acute inpatient rehab hospital), 28 days (skilled nursing facility)] (24, 25). Although a direct comparison cannot be made, the mean LOS for patients with stroke who were admitted to the rehabilitation wards was 16.8 days (the United States), 22.7 days (China), 24.3 days (Taiwan), 30.8 days (Singapore), 41.7 days (Canada), 54.5–76.3 days (France) (26–31). In Japan, the mean LOS from the time of stroke onset to the home discharge is more than 100 days, which is similar to that of Korea (32).

In addition, there is a lack of service providers who provide stroke rehabilitation services other than those provided in inpatient facilities. The National Health Insurance (NHI) claims data in Korea show that the ratio of inpatient rehabilitation to outpatient rehabilitation or day-patient rehabilitation is 81:19, which means that the proportion of rehabilitation services for patients with stroke in inpatient facilities is overwhelmingly high (33).

### Provision of Transitional Rehabilitation and Chronic Long-Term Care Services

Transfer is based on the patient's decision rather than systematically transferring patients according to the post-onset period or severity. Within the NHI system, patients can receive specialized rehabilitation treatment for up to 2 h per day (1 h for physical therapy and another 1 h for occupational therapy) regardless of the type of medical institution for up to 2 years after stroke onset. If the patient wants to receive stroke rehabilitation services in acute care hospitals, it is also possible to transfer from an acute or subacute care unit to another acute care facility. However, it is common to transfer the patient with stroke to a rehabilitation facility after acute stroke care because if a patient's hospitalization period is longer than the period set for each type of medical institution, the transfer is induced indirectly by deducting the patient's hospitalization fee.

While NHI recognizes the services provided by medical institutions as benefits to patients with stroke, long-term care insurance (LTCI) provides benefits such as physical activity or housework support through home-based and institution-based benefits, and special benefits in cash when patients with stroke need to receive care from their family caregivers (34). The LTCI, like NHI, is based on the social insurance system, and the finances of LTCI operate separately from the NHI. To be covered by LTCI, the patient has to be approved by the LTCI eligibility assessment committee. The introduction of LTCI reduced the burden of medical costs by rationalizing long-term health care utilization (35).

The distribution of institutions that provide rehabilitation services is uneven in Korea. Under such circumstances, a system in which a patient with a stroke can flexibly choose a rehabilitation facility has the advantage of receiving services according to the distribution and characteristics of medical resources in each region. The availability of a free selection of medical institutions also has the advantage of reflecting the preferences of medical consumers as much as possible.

Compared to general hospitals, LTCHs have less disincentive for long-term hospitalization and lack community- or home-based care and rehabilitation services after discharge. As a result, the number of inpatients in long-term care hospitals has increased steadily and tends to lead to long-term hospitalization (36).

LTCHs are rehabilitation service providers for patients with stroke. LTCHs have hospital beds to provide medical services for patients who need long-term hospitalization and are classified as hospitals along the same lines as general hospitals. LTCHs in Korea are required to provide inpatient subacute care to patients with geriatric diseases, chronic illnesses, and those who are recovering from surgery or other injuries, including stroke (37). Instead of a fee-for-service reimbursement method, which is a general payment model for NHI in Korea, LTCHs are reimbursed by the daily charge or per diem fee, depending on the physical or cognitive impairment, severity of behavioral problems, and functional status of patients with stroke. Some items, including specialized rehabilitation treatment, can be paid according to the fee-for-service method.

In Korea, LTCH resources only comprised 12,202 hospital beds in 92 institutions in 2005 but increased about 20 times to 268,084 beds in 1,472 institutions in 2020 (38). Considering that the total number of hospital beds in Korea has doubled over the same period, the increase in the number of beds in LTCHs is highly significant.

Community-based rehabilitation (CBR) has been implemented to provide long-term care for community-dwelling patients with disability (39). To support CBR, fourteen “Regional Health and Medical Center for Persons with Disability” have been established nationwide and the “Physician for the Disabled” system (40). The specific program for long-term rehabilitative care for stroke survivors has been provided under the CBR program, to the legally registered persons with disabilities and preliminary persons with disabilities, only for those who voluntarily visited public health centers. Nevertheless, generalized rehabilitation service in the community setting is yet to be established.

### Medical Rehabilitation Delivery System

Comprehensive rehabilitation services for patients with stroke should be available along the continuum of care from the acute stage to the subacute and chronic phases. In general, after completing acute stroke treatment, medical staff, patients, and family caregivers determine whether to discharge the patient or transfer them to a comprehensive inpatient or outpatient rehabilitation facility based on the availability and affordability of informal or formal caregivers and the patient's functional status. When transferring a patient with stroke from a hospital to a rehabilitation facility, the patient and family caregivers must decide on the rehabilitation institution. However, there is no comprehensive or standardized approach to implement transition management protocols that consider patients' severity or medical needs during post-stroke rehabilitation in Korea.

If there is a discharge care team in an acute-stage medical institution, patients with stroke and their families receive help in transferring medical records and collecting hospital information. However, since the establishment of a discharge care team is not the duty of the medical institution, not all patients and caregivers receive systematic and uniform information in the triage scheme process.

For chronic stroke patients, researchers have made several attempts to assess the long-term disabilities and rehabilitative needs of the stroke patients (41, 42). However, there is no standardized assessment method in long-term stroke survivors supported by NHI or LTCI. Currently, the assessment of disability and rehabilitative planning in long-term stroke survivors are performed individually by the doctors.

## Challenges and Future Perspectives

Considering the status of the service delivery system for stroke rehabilitation in Korea described above, the problems of transitional and long-term care rehabilitation are as follows. First, there is still a lack of differentiation and designation of specialized medical rehabilitation institutions that will be in charge of the transitional phase and patient discharge. Second, patients are hospitalized for a long time in LTCHs because of a lack of outpatient rehabilitation services and home-based services in the local community and a bias toward inpatient rehabilitation treatment rather than outpatient services. Finally, the transfer for post-stroke rehabilitation or discharge after acute stroke treatment is not planned systematically.

**Figure 2:**
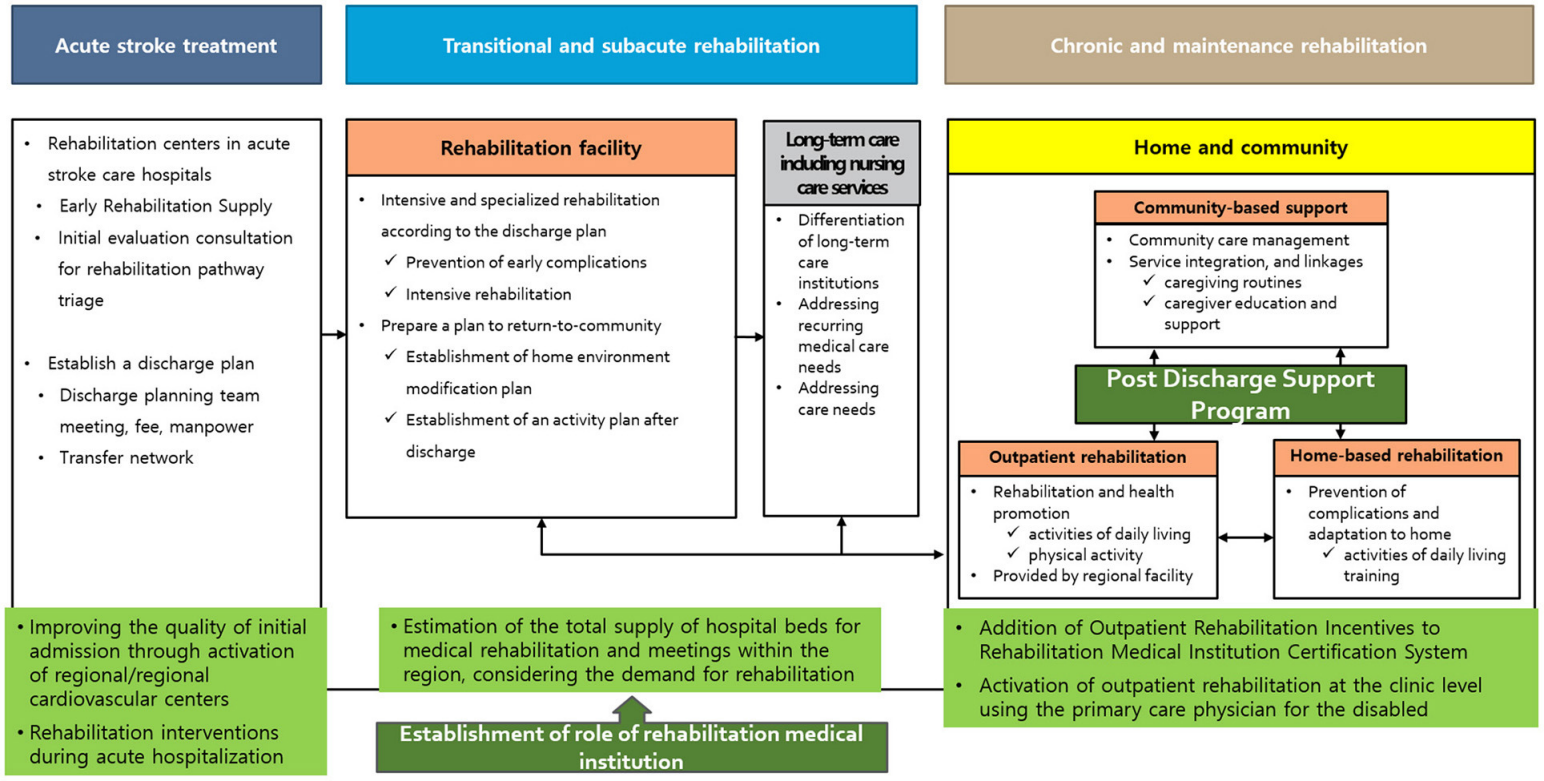
Model of the medical rehabilitation supply system [adopted from (21)].

The Korean government has undertaken three pilot projects and plans outlined below (Figure 2) to solve this problem.

### Designation of Medical Rehabilitation Institutions and Continuous Management of Outcome Indicators

Since 2020, hospitals with a certain level of quality have been designated as medical rehabilitation institutions so that intensive rehabilitation treatment is possible during the functional recovery period. A long-term care hospital can be designated as a secondary hospital after the conversion. The plan is to simultaneously increase the amount of rehabilitation treatment provided and incentives for the fee in these designated hospitals and recognize the fee related to discharge. In addition, pilot projects related to rehabilitation and social return centered on these medical rehabilitation institutions are planned. The outcome is scheduled to be evaluated and re-designated every 3 years, and by 2025, the project is planned to expand these resources to include 25,000 beds in 150 hospitals (43).

### Activation of Outpatient Rehabilitation Service, Development of Home-Based Rehabilitation Service, and Designation of a Physician for the Disabled

Since 2019, the “Physician for the Disabled” system that treats patients with disabilities in the chronic phase, including stroke survivors, has been implemented, and incentives for counseling and treatment are provided to medical staff. After the COVID-19 pandemic, the number of care plans has increased in the pilot project for doctors who treat patients with disabilities. Moreover, non-face-to-face patient management services have been established and operated to facilitate home visitation. Lastly, standardized assessments of long-term disability and rehabilitative needs and planning of rehabilitative management are warranted to reduce the burden of disability in survivors with chronic stroke.

### Operation of a Pilot Project to Establish a Rehabilitation Delivery System

A pilot project was started in 2021 to comprehensively evaluate the patient's condition on discharge from a general hospital-level medical institution to establish a system that enables a smooth return to the local community through an appropriate discharge plan. By designating representative tertiary hospitals, medical rehabilitation institutions, and long-term care hospitals in 19 districts in Korea, the patient linkage process was operationalized and service fees were determined for all processes such as consultation, conferences, and education.

## Conclusion

Stroke care and rehabilitation requires a well-organized comprehensive model from acute stage to chronic stage with a standardized delivery system and proper allocation of medical resources. The current Korean medical practice for stroke rehabilitation is focused on inpatient rehabilitation and lacks the use of a systematic transitional care model including standardized triage system and relay of patients from acute inpatient rehabilitation to chronic community-based rehabilitation. Further, insufficient long-term community and outpatient rehabilitation resources contributes to unmet needs during long-term rehabilitation and care for patients with stroke. The Korean government and specialists are trying to address these problems by establishing standardized rehabilitation delivery system, developing and monitoring the outcome indicators of rehabilitative management and developing pilot projects to promote outpatient or community-based rehabilitation.

## Author Contributions

All authors listed have made a substantial, direct, and intellectual contribution to the work and approved it for publication.

## Funding

This work was supported by the Research Program funded by the Korea National Institute of Health (2020ER630602), Rehabilitation Research & Development Support Program (#NRC RSP-2019017), National Rehabilitation Center, Ministry of Health and Welfare, Korea.

## Conflict of Interest

The authors declare that the research was conducted in the absence of any commercial or financial relationships that could be construed as a potential conflict of interest.

## Publisher's Note

All claims expressed in this article are solely those of the authors and do not necessarily represent those of their affiliated organizations, or those of the publisher, the editors and the reviewers. Any product that may be evaluated in this article, or claim that may be made by its manufacturer, is not guaranteed or endorsed by the publisher.

## References

[1] Kim JY Kang K Kang J Koo J Kim DH Kim BJ. Executive summary of stroke statistics in Korea 2018: a report from the epidemiology research council of the Korean stroke society. J Stroke. (2019) 21:42–59. 10.5853/jos.2018.03125 30558400 PMC6372894

[2] Kim SE Lee H Kim JY Lee KJ Kang J Kim BJ. Three-month modified rankin scale as a determinant of 5-year cumulative costs after ischemic stroke: an analysis of 11,136 patients in Korea. Neurology. (2020) 94:e978–91. 10.1212/WNL.0000000000011295 32029544

[3] Kwon S Park JH Kim WS Han K Lee Y Paik NJ. Health-related quality of life and related factors in stroke survivors: data from Korea National Health and Nutrition Examination Survey (KNHANES) 2008 to 2014. PLoS ONE. (2018) 13:e0195713. 10.1371/journal.pone.0195713 29634768 PMC5892928

[4] Jang MU Kang J Kim BJ Hong JH Yeo MJ Han MK. In-hospital and post-discharge recovery after acute ischemic stroke: a nationwide multicenter stroke registry-base study. J Korean Med Sci. (2019) 34:e240. 10.3346/jkms.2019.34.e240 31538419 PMC6753366

[5] Kim DY Kim Y-H Lee J Chang WH Kim M-W Pyun S-B. Clinical practice guideline for stroke rehabilitation in Korea 2016. Brain Neurorehabil. (2017) 10(Suppl 1). 10.12786/bn.2017.10.e11 PMC12710289 41415589

[6] Chang WH Sohn MK Lee J Kim DY Lee S-G Shin Y-I. Role of intensive inpatient rehabilitation for prevention of disability after stroke: the Korean Stroke Cohort for Functioning and Rehabilitation (KOSCO) Study. Brain Neurorehabil. (2016) 9:e4. 10.12786/bn.2016.9.e4 PMC9833474 36743198

[7] Winters C Kwakkel G van Wegen EEH Nijland RHM Veerbeek JM Meskers CGM. Moving stroke rehabilitation forward: the need to change research. NeuroRehabilitation. (2018) 43:19–30. 10.3233/NRE-172393 30056434

[8] Horgan NF O'Regan M Cunningham CJ Finn AM. Recovery after stroke: a 1-year profile. Disabil Rehabil. (2009) 31:831–9. 10.1080/09638280802355072 19093275

[9] Im HW Kim WS Kim S Paik NJ. Prevalence of worsening problems using post-stroke checklist and associations with quality of life in patients with stroke. J Stroke Cerebrovasc Dis. (2020) 29:105406. 10.1016/j.jstrokecerebrovasdis.2020.105406 33254377

[10] Ustün TB Chatterji S Bickenbach J Kostanjsek N Schneider M. The international classification of functioning, disability and health: a new tool for understanding disability and health. Disabil Rehabil. (2003) 25:565–71. 10.1080/0963828031000137063 12959329

[11] Chen T Zhang B Deng Y Fan JC Zhang L Song F. Long-term unmet needs after stroke: systematic review of evidence from survey studies. BMJ Open. (2019) 9:e028137. 10.1136/bmjopen-2018-028137 31110106 PMC6530326

[12] Ullberg T Zia E Petersson J Norrving B. Perceived unmet rehabilitation needs 1 year after stroke: an observational study from the Swedish stroke register. Stroke. (2016) 47:539–41. 10.1161/STROKEAHA.115.011670 26732564

[13] Unit TEI. The Cost of Inaction:Secondary Prevention of Cardiovascular Disease in Asia-Pacific. Unit TEI (2020).

[14] Kim J Hwang YH Kim JT Choi NC Kang SY Cha JK. Establishment of government-initiated comprehensive stroke centers for acute ischemic stroke management in South Korea. Stroke. (2014) 45:2391–6. 10.1161/STROKEAHA.114.006134 24994720

[15] Act on the Prevention and Management of Cardio-Cerebrovascular Diseases (Act No. 14217) (2016).

[16] Ministry of Health and Welfare. The Comprehensive Management Plan for Cardiocerebrovascular Disease from 2018 to 2022. Ministry of Health and Welfare (2018).

[17] Joa KL Han TR Pyun SB Rah UW Park JH Kim YH. Inpatient stroke rehabilitation outcomes in Korea derived from the Korean brain rehabilitation centers' online database system for the years 2007 to 2011. J Korean Med Sci. (2015) 30:644–50. 10.3346/jkms.2015.30.5.644 25931798 PMC4414651

[18] Ministry of Health and Welfare. Regional Cardiocerebrovascular Center. Ministry of Health and Welfare (2018).

[19] Health Insurance Review & Assessment Service. The Report for 8th Acute Stroke Care Quality Assessment. Health Insurance Review & Assessment Service (2020).

[20] Kim WH Park YG Shin HI Im SH. The world report on disability and recent developments in South Korea. Am J Phys Med Rehabil. (2014) 93(Suppl. 1):S58–62. 10.1097/PHM.0000000000000024 24356084

[21] National Health Insurance Service. Developing a model for an improved medical care supply system. (2019). Available online at: http://www.alio.go.kr/download.dn?fileNo=2514663 (accessed July 11, 2021) (Korean).

[22] Kang JH Bae HJ Choi YA Lee SH Shin HI. Length of hospital stay after stroke: a Korean nationwide study. Ann Rehabil Med. (2016) 40:675–81. 10.5535/arm.2016.40.4.675 27606274 PMC5012979

[23] Jung SH Lee KM Park SW Chun MH Jung HY Kim IS. Inpatient course and length of hospital stay in patients with brain disorders in South Korea: a population-based registry study. Ann Rehabil Med. (2012) 36:609–17. 10.5535/arm.2012.36.5.609 23185724 PMC3503935

[24] García-Rudolph A Cegarra B Opisso E Tormos JM Bernabeu M Saurí J. Predicting length of stay in patients admitted to stroke rehabilitation with severe and moderate levels of functional impairments. Medicine. (2020) 99:e22423. 10.1097/MD.0000000000022423 33120737 PMC7581132

[25] Main Line Health. Acute Inpatient Rehab Hospital vs. Skilled Physician for the Disabled Facility Available online at: https://www.mainlinehealth.org/specialties/rehab/inpatient/snf-vs-acute-rehab (accessed September 27, 2021).

[26] Camicia M Wang H DiVita M Mix J Niewczyk P. Length of stay at inpatient rehabilitation facility and stroke patient outcomes. Rehabil Nurs. (2016) 41:78–90. 10.1002/rnj.218 26009865

[27] Zhang X Qiu H Liu S Li J Zhou M. Prediction of prolonged length of stay for stroke patients on admission for inpatient rehabilitation based on the international classification of functioning, disability, and health (ICF) generic set: a study from 50 centers in China. Med Sci Monit. (2020) 26:e918811. 10.12659/MSM.918811 31901931 PMC6977619

[28] Hung CY Wu WT Chang KV Wang TG Han DS. Predicting the length of hospital stay of post-acute care patients in Taiwan using the Chinese version of the continuity assessment record and evaluation item set. PLoS ONE. (2017) 12:e0183612. 10.1371/journal.pone.0183612 28832680 PMC5568231

[29] Tan WSM Heng BHMMF Chua KS Chan KF. Factors predicting inpatient rehabilitation length of stay of acute stroke patients in Singapore. Arch Phys Med Rehabil. (2009) 90:1202–7. 10.1016/j.apmr.2009.01.027 19577034

[30] Grant CMD Goldsmith CHP Anton HA. Inpatient stroke rehabilitation lengths of stay in Canada derived from the national rehabilitation reporting system, 2008 and 2009. Arch Phys Med Rehabil. (2014) 95:74–8. 10.1016/j.apmr.2013.08.014 24001444

[31] Cassoudesalle H Nozères A Petit H Cressot V Muller F Rouanet F. Post-acute referral of stroke victims in a French urban area: results of a specific program. Ann Phys Rehabil Med. (2016) 59:248–54. 10.1016/j.rehab.2016.02.003 27009910

[32] Murata K Hinotsu S Sadamasa N Yoshida K Yamagata S Asari S. Healthcare resource utilization and clinical outcomes associated with acute care and inpatient rehabilitation of stroke patients in Japan. Int J Qual Health Care. (2017) 29:26–31. 10.1093/intqhc/mzw127 27979962

[33] National Health Insurance Service. Reform strategies for healthcare delivery system in Korea. (2020). Available online at: http://www.alio.go.kr/download.dn?fileNo=2607762 (accessed July 11, 2021) (Korean).

[34] National Health Insurance Service. Insurance benefit of LTCI. Available online at: https://www.nhis.or.kr/static/html/wbd/g/a/wbdga0503.html (accessed July 11, 2021).

[35] Kim SH Kim DH Kim WS. Long-term care needs of the elderly in Korea and elderly long-term care insurance. Soc Work Public Health. (2010) 25:176–84. 10.1080/19371910903116979 20391260

[36] Choi JW Park EC Lee SG Park S Ryu HG Kim TH. Does long-term care insurance reduce the burden of medical costs? A retrospective elderly cohort study. Geriatr Gerontol Int. (2018) 18:1641–6. 10.1111/ggi.13536 30311345

[37] Ga H. Long-term care system in Korea. Ann Geriatr Med Res. (2020) 24:181–6. 10.4235/agmr.20.0036 32842717 PMC7533195

[38] Ministry of the Interior and Safety. Korea city statistics: the number of medical institutions and hospital beds. Available online at: https://kosis.kr/statisticsList/statisticsListIndex.do?parentId=B.1&vwcd=MT_ZTITLE&menuId=M_01_01#content-group (accessed July 11, 2021) (Korean).

[39] Kim YH Jo NK. Community-based rehabilitation in South Korea. Disabil Rehabil. (1999) 21:484–9. 10.1080/096382899297297 10579670

[40] National Rehabilitation Center. Support by Regional Health & Medical Centers for Persons with Disabilities. Available online at: http://www.nrc.go.kr/eng/html/content.do?depth=n_cc&menu_cd=03_06 (accessed January 31, 2022)

[41] Chang WH Sohn MK Lee J Kim DY Lee SG Shin YI. Long-term functional outcomes of patients with very mild stroke: does a NIHSS score of 0 mean no disability? An Interim Analysis of the KOSCO. Study Disabil Rehabil. (2017) 39:904–10. 10.3109/09638288.2016.1170214 27206550

[42] Kim KT Chang WK Jung YS Jee S Sohn MK Ko SH. Unmet needs for rehabilitative management in common health-related problems negatively impact the quality of life of community-dwelling stroke survivors. Front Neurol. (2021) 12:758536. 10.3389/fneur.2021.758536 35002922 PMC8733320

[43] Ministry of Health and Welfare. Medical institution policy: designated as a medical rehabilitation institution. Available online at: http://www.mohw.go.kr/react/policy/index.jsp?PAR_MENU_ID=06&MENU_ID=06290301&PAGE=1&topTitle= (accessed July 11, 2021) (Korean).

